# A large, multicentre, observational, post-marketing surveillance study of the 2:1 formulation of follitropin alfa and lutropin alfa in routine clinical practice for assisted reproductive technology

**DOI:** 10.1186/1477-7827-12-6

**Published:** 2014-01-14

**Authors:** Klaus Bühler, Olaf GJ Naether, Wilma Bilger

**Affiliations:** 1 Centre for Gynaecology, Endocrinology and Reproductive Medicine, Ulm and Stuttgart D-70174, Germany; 2 MVZ Fertility Center Hamburg, Speersort 4, 20095, Hamburg, Germany; 3 Fertility, Endocrinology & General Medicine, Merck Serono GmbH, Alsfelder Str. 17, D-64289, Darmstadt, Germany

**Keywords:** Assisted reproductive technology, Follitropin alfa, Lutropin alfa, Controlled ovarian stimulation, Post-marketing product surveillance

## Abstract

**Background:**

Follicle-stimulating hormone (FSH) and luteinizing hormone (LH) both have a role to play in follicular development during the natural menstrual cycle. LH supplementation during controlled ovarian stimulation (COS) for assisted reproductive technology (ART) is used for patients with hypogonadotropic hypogonadism. However, the use of exogenous LH in COS in normogonadotropic women undergoing ART is the subject of debate. The aim of this study was to investigate characteristics of infertile women who received the 2:1 formulation of follitropin alfa and lutropin alfa (indicated for stimulation of follicular development in women with severe LH and FSH deficiency) in German clinical practice.

**Methods:**

A 3-year, multicentre, open-label, observational/non-interventional, post-marketing surveillance study of women (21-45 years) undergoing ART. Primary endpoint: reason for prescribing the 2:1 formulation of follitropin alfa and lutropin alfa. Secondary variables included: COS duration/dose; oocytes retrieved; fertilization; clinical pregnancy; ovarian hyperstimulation syndrome (OHSS).

**Results:**

In total, 2220 cycles were assessed; at least one reason for prescribing the 2:1 formulation was given in 1834/2220 (82.6%) cycles. Most common reasons were: poor ovarian response (POR) (39.4%), low baseline LH (17.8%), and age (13.8%). COS: mean dose of the 2:1 formulation on first day, 183.1/91.5 IU; mean duration, 10.8 days. In 2173/2220 (97.9%) cycles, human chorionic gonadotrophin was administered. Oocyte pick-up (OPU) was attempted in 2108/2220 (95.0%) cycles; mean (standard deviation) 8.0 (5.4) oocytes retrieved/OPU cycle. Fertilization (≥1 oocyte fertilized) rates: *in vitro* fertilization (IVF), 391/439 (89.1%) cycles; intracytoplasmic sperm injection (ICSI)/IVF + ICSI, 1524/1613 (94.5%) cycles. Clinical pregnancy rate: all cycles, 25.9%; embryo transfer cycles, 31.3%. OHSS: hospitalization for OHSS, 8 (0.36%) cycles, Grade 2, 60 (2.7%), and Grade 3, 1 (0.05%).

**Conclusions:**

In German routine clinical practice, the most common reasons for using the 2:1 formulation of follitropin alfa and lutropin alfa for women undergoing ART were POR, low baseline LH, and age. Severe OHSS incidence was low and similar to that reported previously.

**Trial registration:**

Clinicaltrials.gov NCT01112618

## Background

Follicle-stimulating hormone (FSH) and luteinizing hormone (LH) are involved in the natural menstrual cycle and both have a role to play in follicular development [1]. FSH stimulates follicular development, both LH and FSH are required for oestradiol (E_2_) synthesis, and LH appears to be required for steroidogenesis to occur [1]. In fact, an increase in serum FSH typical of the luteal–follicular transition is a key part of stimulation of ovarian follicle recruitment [2]. In the remaining part of the follicular phase, lower FSH levels mean that no further emergence of immature follicles occurs; at this point, the growth and maturation of the dominant follicle is supported by increases in LH levels [2].

Women with abnormally low levels of LH and FSH caused by severely reduced hypothalamic or pituitary activity are classified as having World Health Organization Group I anovulatory infertility or hypogonadotropic hypogonadism (HH) [3,4]. It is generally considered that LH supplementation during controlled ovarian stimulation (COS) for assisted reproductive technology (ART) should be used in patients with HH [1,4,5]. However, there has been considerable debate over the use of exogenous LH in COS in normogonadotropic women undergoing ART [1,2,6]. The use of long gonadotrophin-releasing hormone agonist protocols causes profound pituitary suppression, which results in reduced LH levels that are similar to those that characterize HH (for which exogenous LH supplementation is recommended) [1]. Currently, studies investigating the use of exogenous LH for COS in normogonadotropic women have yielded conflicting results [1]. However, there is evidence to suggest that specific patient subgroups of normogonadotropic women may benefit from LH supplementation during COS: for example, in women aged ≥35 years and in women with a poor ovarian response [5,7-9]. Also, the use of exogenous LH may be beneficial in women with a hyporesponse to FSH (defined as a normal response in the number of recruited follicles, but requiring an increased and/or prolonged FSH stimulation to continue and complete follicular growth) [10].

Currently, FSH alone is commercially available as urine-derived or recombinant formulations, whereas LH alone is only available as a recombinant formulation. In addition, a 2:1 formulation of recombinant human (r-h)FSH and r-hLH (in a fixed-dose combination of 150 IU r-hFSH [follitropin alfa]:75 IU r-hLH [lutropin alfa]) has been developed [11-13]. This 2:1 formulation of follitropin alfa and lutropin alfa is indicated for the stimulation of follicular development in women with severe LH and FSH deficiency (defined by an endogenous serum LH level of <1.2 IU/L) [14] and has been available in Germany since October 2007.

To investigate the characteristics of women who received the 2:1 formulation of follitropin alfa and lutropin alfa in routine clinical practice in Germany, a 3-year post-marketing surveillance study was initiated. The study explored the reasons why physicians chose to treat women with the 2:1 formulation of follitropin alfa and lutropin alfa, as well as how they defined the characteristics of patients who may benefit from supplemental LH in daily practice. An interim analysis of data from 919 ART cycles collected between January and November 2008 has been reported previously [3]. In the current report, we present ART treatment cycle data for the full 3 years.

## Methods

ART procedures were conducted according to standard practice in Germany at the time of the study – embryo selection was not permitted, no more than three embryos could be transferred, and supernumerary two-pronuclear (2PN) oocytes could be cryopreserved for future use [15].

### Study design

This was a 3-year, multicentre, open-label, observational (non-interventional), post-marketing surveillance study (Clinicaltrials.gov identifier: NCT01112618; Merck Serono protocol number RH002) that collected data from 45 fertility treatment centres in Germany.

The objective of this study was to investigate the characteristics of women who received treatment with the 2:1 formulation of follitropin alfa and lutropin alfa.

Data were collected from women aged 21–45 years undergoing ART cycles. All patients received the 2:1 formulation of r-hFSH and r-hLH (in a fixed-dose combination of 150 IU r-hFSH [follitropin alfa]:75 IU r-hLH [lutropin alfa], Merck Serono S.A., Geneva, Switzerland, a subsidiary of Merck KGaA, Darmstadt, Germany). Combination treatment with follitropin alfa for dose adaptation was permitted (in accordance with prescribing information) [14]. Cycles involving combination treatment of the 2:1 formulation with clomiphene citrate, human menopausal gonadotrophin, urine-derived FSH, follitropin beta, or lutropin alfa were excluded from the analysis. In addition, cycles were excluded that used frozen 2PN oocytes created during previous cycles.

Only one cycle per patient was to be included in the analysis; if information was available on >1 cycle per patient, data from the earliest cycle were analysed.

### Data collection

All data were recorded anonymously.

Prior to initiation of treatment, the treating physician completed a questionnaire which included a question on the patient’s clinical presentation that led to a diagnosis of severe LH deficiency and, thus, prescribing the 2:1 formulation of follitropin alfa and lutropin alfa. One or more of the following reasons could be selected: low baseline LH level; low baseline E_2_ level; thin endometrium; amenorrhoea; and/or ‘other’. Endometrial thickness prior to COS, baseline E_2_ and LH (on cycle days 2–4) levels, and the presence of amenorrhoea were recorded. Age, body mass index (BMI), antral follicle count (AFC), baseline FSH (cycle days 2–4), and anti-Müllerian hormone (AMH) levels could also be recorded (optional). The questionnaires were sent for data analysis by ANFOMED (ANFOMED GmbH, Möhrendorf, Germany).

In addition, the physician prospectively entered routine clinical and laboratory data for the treatment cycles into a standardized electronic data collection system ‘RecDate’ [16]. The RecDate software system enables the collection, documentation, and evaluation of reproductive medicine data. Clinical pregnancy was recorded; this was defined as transvaginal ultrasound identification of an (intra- or extra-uterine) gestational sac, but also included extrauterine pregnancies or miscarriages confirmed by histological evidence. Follow-up data on pregnancy outcomes were collected for 18 months after the initial study period.

### Outcome measures

The primary outcome measure was the reason for diagnosis of severe LH deficiency and, thus, for prescribing the 2:1 formulation of follitropin alfa and lutropin alfa. Secondary outcome measures relating to the 2:1 formulation of follitropin alfa and lutropin alfa received were: the dose on the first and last days of treatment; whether a consistent dose was used throughout COS; the duration of COS; and the total dose received. Treatment outcome measures included the number of oocytes retrieved, rate of fertilization, and clinical pregnancy rate; available follow-up data were also reported.

Reporting of adverse events and adverse drug reactions is not required in German post-marketing surveillance studies. However, the overall incidence of ovarian hyperstimulation syndrome (OHSS; any Grade) and the incidence of OHSS Grades 2 and 3 were reported. Grade 1 OHSS was considered a normal response to COS.

### Statistical analysis

No calculations of sample size or statistical power were undertaken. No statistical hypotheses were pre-specified. Descriptive statistics were provided (means, standard deviations [SD], frequencies, and percentages). In addition, data for subgroups of patients according to age (≤35 and >35 years) were reported.

## Results

The study was conducted between January 2008 and December 2010. A total of 3825 questionnaires were received from 45 centres in Germany. Following matching with the RecDate database, 2220 ART cycles using the 2:1 formulation of follitropin alfa and lutropin alfa in women aged 21–45 years were identified; these cycles were from 42 centres.

### Patient characteristics

For the 2220 cycles assessed, baseline demographic and clinical characteristics for the overall patient population and according to age group (≤35 years and >35 years) are shown in Table 1. The mean (SD) age of patients was 36.5 (4.00) years, the mean (SD) BMI was 23.1 (3.98), and the mean endometrial thickness was 3.41 (2.45) mm. For the 180 cycles with AMH data reported, the mean (SD) AMH level was 1.7 (2.06) ng/mL; the median AMH level was 1.0 ng/mL (lower and upper quartiles: 0.4 and 2.1 ng/mL). There were 1528/2220 (68.8%) patients aged >35 years. FSH and E_2_ levels at baseline and endometrial thickness were similar in both age groups. However, baseline LH and AMH levels and AFC were higher in patients aged ≤35 years than in the older subgroup of patients.

**Table 1:** Baseline demographic and clinical characteristics

**Characteristic (mean [SD])**	**Age subgroup**		**All patients (*n*** **= 2220)**
	**≤35 years (*n*** **= 692)**	**>35 years (*n*** **= 1528)**	
*Age, years*	31.6 (2.57) (*n =* 692)	38.7 (2.14) (*n =* 1528)	36.5 (4.00) (*n =* 2220)
*BMI, kg/m*^*2*^	23.1 (4.10) (*n =* 683)	23.1 (3.92) (*n =* 1502)	23.1 (3.98) (*n =* 2185)
*FSH level (on cycle days 2–4), IU/L*	10.6 (12.06) (*n =* 209)	10.3 (10.91) (*n =* 395)	10.4 (11.31) (*n =* 604)
*LH level, IU/L*	5.22 (7.92) (*n =* 448)	4.53 (6.57) (*n =* 837)	4.77 (7.07) (*n =* 1285)
*E*_*2*_*level, pg/mL*	42.00 (77.51) (*n =* 431)	43.9 (66.86) (*n =* 814)	43.3 (70.7) (*n =* 1245)
*AMH, ng/mL*	2.1 (3.08) (*n =* 45)	1.5 (1.56) (*n =* 135)	1.7 (2.06) (*n =* 180)
*Endometrial thickness, mm*	3.42 (3.57) (*n =* 344)	3.41 (1.66) (*n =* 717)	3.41 (2.45) (*n =* 1061)
*AFC (number of follicles <11 mm)*	6.8 (5.07) (*n =* 210)	4.5 (3.64) (*n =* 440)	5.3 (4.29) (*n =* 650)

AFC = antral follicle count; AMH = anti-Müllerian hormone; BMI = body mass index; E_2_ = oestradiol; FSH = follicle-stimulating hormone; LH = luteinizing hormone; SD = standard deviation.

### Primary outcome measure

**Figure 1:**
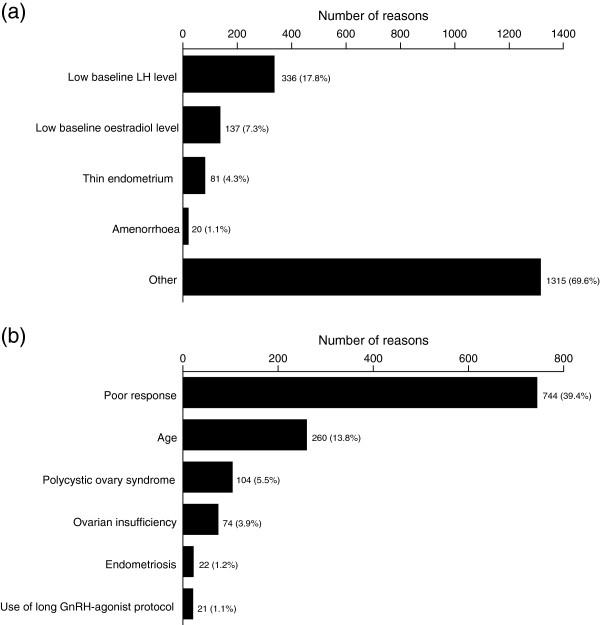
**The reasons cited by physicians for prescribing the 2:1 formulation of follitropin alfa and lutropin alfa for 1834/2220 assisted reproductive technology (ART) cycles. (a)** The number of reported pre-specified reasons (one or more reasons could be given; 1889 reasons were given for 1834 ART cycles); **(b)** Expansion of the pre-specified answer of ‘other’ (1315 responses of other were given in total; the six reasons cited most frequently are shown). Percentages are based on 1889 reasons. GnRH = gonadotrophin-releasing hormone; LH = luteinizing hormone.

At least one reason for prescribing the 2:1 formulation of follitropin alfa and lutropin alfa was given for 1834/2220 (82.6%) cycles (Figure 1). Physicians gave one pre-specified reason in 1780 cycles and more than one pre-specified reason in 54 cycles (Figure 1a). The pre-specified answer of ‘other’ was analysed further by ‘actual reason given’ (Figure 1b). A total of 1889 reasons (1834 cycles) were stated and the four reasons cited most frequently (including the expansion of the category ‘other’) were: poor ovarian response (744/1889; 39.4%); low baseline LH level (336/1889; 17.8%); age (260/1889; 13.8%); and low baseline E_2_ level (137/1889; 7.3%).

### Secondary outcomes

#### COS characteristics

Data on the duration of COS were missing for three cycles. The mean (SD) duration of COS using the 2:1 formulation of follitropin alfa and lutropin alfa was 10.8 (2.90) days (2217 cycles; Table 2). In most (2107/2217; 95.0%) cycles, COS lasted for 6–15 days.

**Table 2:** Controlled ovarian stimulation (COS) characteristics for assisted reproductive technology cycles overall and by age subgroups

**COS variable (mean [SD])**^**a**^	**Age subgroup**		**All patients (*n =*** **2220)**^**b**^
	**≤35 years (*n =*** **692)**	**>35 years (*n =*** **1528)**	
*Duration of COS, days*	11.0 (3.36) (*n =* 690)	10.7 (2.67) (*n =* 1527)	10.8 (2.90) (*n =* 2217)
*Dose*^*c*^*on first day of COS, IU*	163.1/81.6 [= 244.7] (65.37/32.68 [= 98.05]) (*n =* 567)	192.0/96.02 [= 288.1] (80.71/40.36 [= 121.07]) (*n =*1258)	183.1/91.5 [= 274.6] (77. 4/38.7 [= 116.1]) (*n =* 1825)
*Dose*^*c*^*on last day of COS, IU*	168.11/84.06 [= 252.17] (71.12/35.56 [= 106.68]) (*n =* 531)	187.89/93.95 [= 281.84] (85.99/42.99 [= 128.98]) (*n =* 1199)	181.8/90.9 [= 272.7] (82. 2/41.1 [= 123.3]) (*n =* 1730)
*Total dose*^*c*^*required, IU*	1617.2/808.6 [= 2425.8] (995.3/497.6 [= 1492.9]) (*n =* 648)	2064.2/1032.1 [= 3096.3] (1115.4/557.7 [= 1673.1]) (*n =* 1409)	1923.4/961.7 [= 2885.1] (1098.6/549.3 [= 1647.9]) (*n =* 2057)

^a^The number of cycles for which each COS variable was reported varied.

^b^The total number of cycles reported in this study was 2220; however, the duration of COS was unavailable for three cycles.

^c^Dose of the 2:1 formulation of follitropin alfa and lutropin alfa.

SD = standard deviation.

Similar mean doses of the 2:1 formulation of follitropin alfa and lutropin alfa were used on the first and last day of COS: 183.1/91.5 IU and 181.8/90.9 IU, respectively. The data according to age subgroup showed that lower mean doses of the 2:1 formulation of follitropin alfa and lutropin alfa were used (on the first and last days of COS and in total) in patients aged ≤35 years than in older patients (Table 2).

In most (2076/2220; 93.5%) cycles, the 2:1 formulation of follitropin alfa and lutropin alfa was the only treatment used for COS. Dose adaptation using follitropin alfa was reported in 144/2220 (6.5%) cycles. The mean (SD) dose of additional follitropin alfa was 1982.7 (1038.4) IU. Data on dose were available for 2074 cycles, in which only the 2:1 formulation of follitropin alfa and lutropin alfa was used for COS. In these cycles, most (1875/2074; 90.4%) used a consistent dose throughout COS: one vial (150/75 IU) in 908/2074 cycles; two vials (300/150 IU) in 538/2074 cycles; three vials (450/225 IU) in 324/2074 cycles; and other doses in 105/2074 cycles. The dose of the 2:1 formulation of follitropin alfa and lutropin alfa was adjusted during treatment in 199/2074 cycles. The dose was increased in 99 patients, decreased in 76, and only temporarily changed in 24 patients.

#### Treatment outcomes

In 2173/2220 (97.9%) cycles, human chorionic gonadotrophin was administered to trigger ovulation, and in 2108/2220 (95.0%) cycles, oocyte pick-up (OPU) was attempted (Table 3). A mean (SD) number of 8.0 (5.40) oocytes were retrieved per OPU cycle (Table 3). A slightly higher mean number of oocytes were retrieved per OPU cycle in patients aged ≤35 years (*n* = 670) than in patients aged >35 years (*n* = 1438): 9.3 (5.85) versus 7.4 (5.07) oocytes, respectively, although the difference was not analysed statistically.

**Table 3:** Oocyte pick-up (OPU), embryo transfer (ET), and pregnancy rates for assisted reproductive technology cycles

**Outcome**	**Age subgroup**		**All patients**
	**≤35 years**	**>35 years**	
*hCG administered,* n *(%)*	679 (98.1) (*n =* 692)	1494 (97.8) (*n =* 1528)	2173 (97.9) (*n =* 2220)
*OPU attempted,* n *(%)*	670 (96.8)(*n =* 692)	1438 (94.1) (*n =* 1528)	2108 (95.0) (*n =* 2220)
*Number of oocytes retrieved, mean (SD)*^*a*^	9.3 (5.85) (*n =* 670)	7.4 (5.07) (*n =* 1438)	8.0 (5.40) (*n =* 2108)
*ET performed,* n *(%)*	617 (89.2) (*n =* 692)	1215 (79.5) (*n =* 1528)	1832 (82.5) (*n =* 2220)^b^
*Number of embryos transferred, mean (SD)*^*c*^	2.0 (0.51) (*n =* 617)	2.0 (0.65) (*n =* 1215)	2.0 (0.60) (*n =* 1832)
*Implantation achieved,* n *(%)*^*d*^	346 (29.3) (*n =* 1179)	405 (17.4) (*n =* 2323)	751 (21.4) (*n =* 3502)
*Clinical pregnancy per ET cycle,* n *(%)*	255 (41.3) (*n =* 617)	319 (26.26) (*n =* 1215)	574 (31.3) (*n =* 1832)

The study evaluated data for 2220 assisted reproductive technology cycles; the percentages given in the table were calculated from different denominators as indicated.

^a^OPU cycles.

^b^ET was not performed in 388 cycles.

^c^ET cycles.

^d^Implantation rate is defined as the number of (intra- and extra-uterine) gestational sacs/number of transferred embryos.

hCH = human chorionic gonadotrophin; SD = standard deviation.

The embryo transfer (ET) rate, number of embryos transferred, and implantation rate are presented in Table 3.

*In vitro* fertilization (IVF) alone was used in 439 cycles, intracytoplasmic sperm injection (ICSI; or a combination of IVF and ICSI) was used in 1613 cycles. Fertilization of at least one oocyte was achieved in 391/439 (89.1%) IVF cycles and in 1524/1613 (94.5%) ICSI (or a combination of IVF and ICSI) cycles.

#### Clinical pregnancy and follow-up

Clinical pregnancy was achieved in 574 cycles (25.9% of all cycles or 31.3% of ET cycles). The clinical pregnancy rate per ET cycle was 41.3% in patients aged ≤35 years and 26.3% in those aged >35 years.

Eighteen-month follow-up data were available for 458 patients. A total of 318 pregnancies resulted in live births: 258 singleton live births; 56 twins; and 4 resulted in higher multiple births.

There were 140 miscarriages; the miscarriage rate per reported clinical pregnancy was 24.4% (140/574), or using the follow-up dataset, the miscarriage rate was 30.6% (140/458). The baseline characteristics for patients who had a clinical pregnancy and experienced a miscarriage are shown in Table 4.

**Table 4:** Key baseline characteristics for the ‘miscarriage’ and ‘no miscarriage’ subgroups who had a clinical pregnancy

**Baseline characteristic**	**Miscarriage**	**No miscarriage**
*Age, years*	36.0 (4.35) (*n =* 140)	34.9 (3.88) (*n =* 434)
*FSH level, IU/L*	9.5 (11.50) (*n =* 34)	10.1 (12.41) (*n =* 114)
*LH level, IU/L*	3.9 (5.09) (*n =* 83)	4.2 (7.79) (*n =* 268)
*AMH level, ng/mL*	2.8 (3.90) (*n =* 10)	1.9 (1.48) (*n =* 32)

In total, there were 574 women in the ‘miscarriage’ and ‘no miscarriage’ subgroups who had a clinical pregnancy.

All values are mean (standard deviation).

AMH = anti-Müllerian hormone; FSH = follicle-stimulating hormone; LH = luteinizing hormone.

There were fewer miscarriages in patients aged ≤35 years (51/255, 20.0% per reported clinical pregnancy; 51/198, 25.8% follow-up dataset) than in patients aged >35 years (89/319, 27.9% per reported clinical pregnancy; 89/260, 34.2% follow-up dataset).

#### Ovarian hyperstimulation syndrome

OHSS (of any grade) was reported in 337/2220 (15.2%) cycles, hospitalization due to OHSS in 8 (0.36%) cycles, and Grade 1, Grade 2 and Grade 3 OHSS were reported in 268 (12.1%), 60 (2.7%), and 1 (0.05%) cycles, respectively.

## Discussion

This large (2220 ART cycles), 3-year post-marketing surveillance study provides valuable information on routine clinical practice in Germany regarding the use of the 2:1 formulation of follitropin alfa and lutropin alfa. It is important to investigate how clinicians are using fertility treatments in clinical practice as their prescribing habits may be influenced by evidence in the published literature. The reason cited most frequently for prescribing the 2:1 formulation of follitropin alfa and lutropin alfa in ART cycles was ‘poor ovarian response’ (39% of reported reasons), with low baseline LH level (18%) being the second most frequently cited reason. Age (14%) and low endogenous E_2_ level (7%) were the next most commonly cited reasons.

Information on the dose of the 2:1 formulation of follitropin alfa and lutropin alfa used for COS in ART cycles revealed that in the overall population, the mean first and last doses administered were similar (183.1/91.5 and 181.8/90.9 IU, respectively). In addition, the mean (SD) duration of COS was 10.8 (2.90) days. Furthermore, as expected, higher doses of the 2:1 formulation were used in women aged >35 years than in the younger age group. The rate of started cycles where ET was not performed in this study (388/2220; 17.5%) was similar to that reported in the 2010 German IVF Registry (64,348/75,928 plausible cycles; ~15%) [17].

The use of the 2:1 formulation of follitropin alfa and lutropin alfa for COS during routine clinical practice was effective in achieving pregnancies in ART cycles, with a clinical pregnancy rate per ET cycle of 31.3%. In the two age groups studied, clinical pregnancy rate per ET cycle was markedly different, with the rate being lower in older women as might be expected. The German IVF Registry reports annual data on women undergoing ART [15,17,18]. The clinical pregnancy rates reported here for women aged ≤35 years (41.3%) and >35 years (26.3%) are similar to those from prospectively collected cycles reported in the German IVF Registry (2008–2010): clinical pregnancy rates (per ET cycle) of 34.2–39.5% for women aged ≤34 years and 13.7–28.9% for women aged ≥35 years.

Approximately 70% of the current study population was aged ≥35 years. This might be expected as there is a general trend for delayed childbearing in Western societies [19], with many couples seeking ART for this reason [20]. However, although a recent study has failed to find differences in outcomes for women ≥35 years treated with or without LH supplementation during COS [21], there are a growing number of studies that have demonstrated a benefit from LH supplementation [5], which may have contributed to older patients being prescribed the 2:1 formulation of follitropin alfa and lutropin alfa and consequently entering this study. For example, in a recent meta-analysis of data from patients aged ≥35 years (7 randomized controlled trials; 902 ART cycles) r-hLH supplementation of r-hFSH for COS resulted in higher implantation (odds ratio [OR]: 1.36; 95% confidence interval [CI]: 1.05, 1.78) and clinical pregnancy (OR: 1.37; 95% CI: 1.03, 1.83) rates than r-hFSH alone [22].

The rate of miscarriage reported in this study (24.4%) was slightly higher than those reported in the German IVF Registry in 2009 and 2010 (18.4–18.7% for fresh ET and 23.3% for frozen ET [calculated per clinical pregnancy; all ages combined]) [15,17]. However, it should be kept in mind that a large proportion of patients in the current study were aged ≥35 years, and advanced maternal age has been linked to a greater risk of foetal loss [19,23].

Our study indicates that the 2:1 formulation of follitropin alfa and lutropin alfa has a favourable profile in terms of OHSS (1 case of Grade 3 OHSS [0.05% cycles] and 8 cases of OHSS requiring hospitalization [0.36% cycles]). This finding is supported by previous data on the 2:1 formulation of follitropin alfa and lutropin alfa [14]. Furthermore, the rate of Grade 3 OHSS reported here is lower than that reported in the German IVF Registry in 2009 (0.27%) and 2010 (0.23%) per stimulation protocol (*n* = 41,925 and *n* = 41,102, respectively) [15,17]. However, the low incidence of OHSS reported in the present study may be expected from the population of patients studied as they were likely to have a low ovarian response.

The key strength of this observational study is the use of the RecDate database, which provided prospective documentation of treatment cycles at approximately 85% of all German fertility centres during 2008 to 2010 [6]. Thus, our findings may be considered to reflect normal clinical ART practice in Germany and, therefore, offer valuable insights into the use of the 2:1 formulation of follitropin alfa and lutropin alfa. Furthermore, the large number of observations included in this investigation were collected without the strict inclusion or exclusion criteria that are mandatory in a randomized controlled trial or meta-analysis and so are likely to reflect routine clinical practice more accurately.

We fully acknowledge the observational nature of this post-marketing surveillance study and the intrinsic limitations of such a study design. This type of study relies on the reporting of data under less stringent conditions than those in a randomized clinical trial. As such, some data sets in the current study are incomplete (and the number of patients with evaluable data was different for each outcome). Furthermore, there is the potential for variability in responses submitted by physicians to the questionnaire, as responses depended on the clinical opinion of each physician. For example, individual physicians may have used a different definition of ‘poor ovarian response’, such as patients who had a poor response to previous cycles or those with an expected poor response for other reasons. A standardized definition of a poor response to COS was published in 2011 [24] after the current study had been completed.

In summary, the three reasons cited most frequently for the use of the 2:1 formulation of follitropin alfa and lutropin alfa were poor ovarian response, low baseline LH level, and age. The pregnancy rates reported here using the 2:1 formulation of follitropin alfa and lutropin alfa were similar to those reported in the German IVF Registry during the same time frame; the incidence of severe OHSS reported in this study was low and similar to that reported previously.

## Abbreviations

AFC: Antral follicle count; AMH: Anti-Müllerian hormone; ART: Assisted reproductive technology; BMI: Body mass index; COS: Controlled ovarian stimulation; E2: Oestradiol; ET: Embryo transfer; FSH: Follicle-stimulating hormone; hCH: Human chorionic gonadotrophin; HH: Hypogonadotropic hypogonadism; ICSI: Intracytoplasmic sperm injection; IVF: *In vitro* fertilization; LH: Luteinizing hormone; OHSS: Ovarian hyperstimulation syndrome; OPU: Oocyte pick-up; POR: Poor ovarian response; 2PN: 2 pro-nuclear; SD: Standard deviation.

## Competing interests

KB and OGJN have no conflicts of interest. WB is an employee of Merck Serono GmbH, Germany.

## Authors’ contributions

All authors contributed to the study design, data analysis, manuscript drafting and critical discussion and have read and approved the final manuscript.
